# Measuring and Assessing Healthcare Organisational Culture in the England’s National Health Service: A Snapshot of Current Tools and Tool Use

**DOI:** 10.3390/healthcare7040127

**Published:** 2019-11-01

**Authors:** Dominic Simpson, Sharon Hamilton, Robert McSherry, Rebecca McIntosh

**Affiliations:** 1 School of Health and Life Sciences, Teesside University, Middlesbrough TS1 3BX, UK; sharon.hamilton@tees.ac.uk; 2 Teesside Centre for Evidence-Informed Practice: A JBI Centre of Excellence Middlesbrough TS1 3BX, UK; 3 NHS Calderdale Clinical Commissioning Group, Halifax HX3 5AX, UK; robert.mc1527@gmail.com; 4 Cardiac Intensive Care, South Tees NHS Foundation Trust, Middlesbrough TS4 3BW, UK; rebeccamcintosh@hotmail.co.uk

**Keywords:** organisational culture, healthcare culture, NHS, patient safety culture, defining culture, clinical governance, patient safety measurement, measuring culture

## Abstract

Healthcare Organisational Culture (OC) is a major contributing factor in serious failings in healthcare delivery. Despite an increased awareness of the impact that OC is having on patient care, there is no universally accepted way to measure culture in practice. This study was undertaken to provide a snapshot as to how the English National Health Service (NHS) is currently measuring culture. Although the study is based in England, the findings have potential to influence the measurement of healthcare OC internationally. An online survey was sent to 234 NHS hospital trusts, with a response rate of 35%. Respondents who completed the online survey, on behalf of their representative organisations, were senior clinical governance leaders. The findings demonstrate that the majority of organisations, that responded, were actively measuring culture. Significantly, a wide variety of tools were in use, with variable levels of satisfaction and success. The majority of tools had a focus on patient safety, not on understanding the determining factors which impact upon healthcare OC. This paper reports the tools currently used by the respondents. It highlights that there are deficits in these tools that need to be addressed, so that organisations can interpret their own culture in a standardised, evidence-based way.

## 1. Introduction

Across the international healthcare sector, organisational culture (OC) and working environments have become central to all things patient safety. Culture is a term that has become synonymous with patient experience, satisfaction, mortality, and morbidity. Francis [1] chaired an independent public inquiry into catastrophic failings in care at Mid Staffordshire NHS Foundation Trust UK, the inquiry highlighted how healthcare OC was a major contributing factor in the repeated failure to meet patients’ needs. Francis made a total of 290 recommendations based on the official enquiry, one of his recommendations was the need to develop a culture of care barometer, to accurately measure OC in practice.

Over the past 20 years, we have witnessed the rise of research engagement and activities involving stakeholder groups made up; of clinical experts, educational institutions, professional bodies and regulators all of whom are trying to decipher the complexities of the working environment. Understanding local culture has become a primary focus of clinical governance work internationally, there is a recognised urgent need to disentangle the complexity of how healthcare OC is impacting on patient care and staff wellbeing.

The Care Quality Commission (CQC) is the United Kingdom’s (UK) healthcare regulator [2]. The UK Department of Health (DH) is a branch of the UK government concerned with the maintenance of public health and provides leadership for the NHS [3]. The Health Foundation is an independent UK charity that focuses on healthcare improvement by making links between knowledge, research and analysis [4]. Each of these organisations openly acknowledge that cultural change is essential to creating a safe and sustainable NHS which can meet future challenges of increasingly complex patient groups [5,6,7].

This notion of needing to develop a greater understanding of healthcare OC has been echoed in more recent reports such as The Morecambe Bay Investigation, which investigated failings in maternity care at Furness General Hospital UK [8]. The investigation concluded that there were at least seven missed opportunities at almost every level, which meant poor clinical care was not investigated and led to the preventable deaths of one mother and eleven babies [8]. The report highlighted that one of the biggest factors which impacted on these missed opportunities was OC.

Culture was again in the spotlight in the serious case review into failings at Gosport War Memorial Hospital UK. The investigation found an institutionalised culture of practice that accepted the shortening of lives through prescribing and administering opioids without medical justification [9]. Over a period of 12 years, a culture developed where concerns were ignored and unsafe practices went unchallenged, resulting in 456 people having their lives unnecessarily shortened [9,10].

Work by Jones [9], Budd [10], and Kirkup [8] reveals an urgent need to understand how and why these incidents occur and why it is that individual health professionals and teams perform in this way. It is imperative to establish and understand what shapes the basic values, beliefs and assumptions that underpin patterns of this type of behaviour. Similarly, with the Francis inquiry [1], health professionals were found to be neglectful and destructive in their behaviours. These major scandals warrant further analysis by calling for a deeper understanding of the nature of how healthcare OC is assessed and measured in practice.

The measurement and assessment of culture in a healthcare area is not a new idea. The Bristol Inquiry [11] was a landmark inquiry which uncovered an OC that had contributed to the deaths of 35 children. This inquiry sparked national healthcare reform and the introduction of new health legislation. Almost two decades later, despite an increased understanding of the impact culture has on patient mortality, we have seen a plethora of serious case reviews that all find culture as a major determining factor in the deterioration of care [12]. The repeated failings in care demonstrate that we have not yet fully grasped in healthcare how to measure, understand, or change culture.

Mannion, Konteh, and Davies [13] conducted a national survey of tools being used to measure OC in English NHS organisations. This study aims to progress Mannion et al.’s work by establishing a further update on current understanding and awareness of healthcare OC. This is achieved by seeking to answer the following: How are NHS England trusts currently measuring OC in practice, and what tools are available for them for the assessment and measurement of their OC?

### Defining Organisational Culture in the Context of the NHS

OC emerged as a discourse and field of study in the 1980s when a series of popular books about business theory spread the view that to be successful, companies needed to focus on their culture [13]. Culture change was seen as a way to improve productivity and efficiency at work and also as a way of establishing supportive relationships. Davies, Nutley and Mannion, Kufman and McCaught, Hruschka and Hadley, and McSherry and Pearce [14,15,16,17] all generally agree that culture is defined as “The ideas, customs, and social behaviour of a particular people or society” [18]. OC represents the collective values and beliefs of the people who work in the organisation. In healthcare, factors which influence OC are its history, clinical governance, management aims and objectives, and political, economic, technical, and legal dimensions [19]. OC is defined as the way things are done through established attitudes, values, beliefs, and practices which are exhibited within a work environment [20]. These values have been shown to affect the health and wellbeing of employees. Staff values also have a significant impact on the quality and safety of the healthcare delivery along with the working environment [1,21].

## 2. Materials and Methods

### 2.1. Data Collection Instrument

Data collection and analysis comprised of a national electronic survey (via Online Surveys) sent to all English NHS trusts (*N* = 234). The survey, named as part of this study Measurement and Assessment of Organisational Culture Tools online survey (MAOCT), which can be obtained by contacting the corresponding author, was adapted from the questionnaire developed by Mannion et al. [13] The MAOCT was validated by Mannion et al.’s original study.

The 22 question MAOCT was split into three distinct parts. The first sought to agree a definition of culture and understand their clinical governance leader’s interpretation of culture. The second reviewed what tools respondents were currently using in their organisation and the level of satisfaction with their tool of choice. The final set of questions sought to understand what aspects of OC respondents felt were missing from their current tools.

### 2.2. Sample

All English NHS trusts were invited to participate in this study. Individual clinical governance leaders were recruited via their organisations’ research and development departments, and recruitment took place between November 2018 and January 2019. A total of three reminder emails were sent to invited trusts to promote a good response rate. A total of 84 (35%) of those approached responded to the survey. This is a disappointing but not unexpected response rate from an anonymous survey [22,23]. Such a response rate prevents any generilisation of these results, but still allows us to present an indication of current practice in the NHS.

### 2.3. Ethics

This research received ethical approval from Teesside University Research Ethics and Governance Committee and the UK’s Health Research Authority Research Ethics Committee (IRAS ID: 237425).

## 3. Results

### 3.1. Agreeing a Definition

As part of this study we presented clinical governance leaders with our working definition of culture:

‘Culture is co-created through the interactions, communications, influences and collaborations among members of an organisation. In healthcare organisations, this can create cultures within staff groups, shifts, wards, specialities, directorates and whole systems. Within healthcare organisations culture is a reflector of values, ideas, customs and beliefs of that organisation which include quality, safety and compassion in practice. Furthermore, it is these values that distinguish the difference between ‘right’ and ‘wrong’.

We asked clinical governance leaders to what extent does this definition accord with their own understanding of healthcare OC. The result was that 70.9% of respondents strongly agreed with this definition, 23.6% said that they tended to agree. While none of those who responded strongly disagreed, 5.5% of respondents disagreed with this definition; the reason they cited for doing so was the failure to mention leadership in the definition. While we acknowledge that leadership has an impact on defining the culture of an organisation, what we aim to do with this definition is incorporate a level of accountability for staff, not just leaders. Historically, the view has been that the system is inadequate, creating poor culture. However, when culture is defined as being co-created, it makes staff individually and collectively accountable for the system, and thus the culture that they belong to/create.

### 3.2. Tools Currently in Use

Table 1 presents the results in order of popularity, number of trusts using the tool. The mean reported satisfaction score is given. Clinical governance leaders were asked to grade satisfaction with their tool of choice on a scale of 1 to 5, one being not at all satisfied, five being very satisfied. A brief description of the tool was then given. Finally, the tools have been analysed and global themes have been identified. A more detailed description of the global themes is given in Table 2.

The results demonstrate that clinical governance leaders were using different methods to measure culture in their organisations, while a small number of trusts reported using more than one tool (total: 2%), the majority of trusts told us that they were only using one tool.

### 3.3. Aspects of Culture Not Currently Measured

The final part of the MAOCT asked respondents to reflect on areas of OC that they felt their current tool was not capturing. The top three areas of concern for clinical governance leaders were incivility and rudeness (42%), staff morale (19%), and opportunities for whistleblowing and speaking up (16%). The other 23% of responses focused on more specific issues within their trust.

## 4. Discussion

The key findings from this study are that there is no identified standard way to measure culture, that the majority of trusts who responded to this survey are only currently using one tool, and that the current tools in use by respondents do not necessarily capture everything that clinical governance leaders would like them to.

Ideas of culture are also central to quality improvement methods, from basic clinical audit to sustained improvement. There are a wide range of tools to measure culture available on the market, yet it seems that relativity few of these are currently used in the NHS. It is interesting that there was a mixed level of satisfaction in the tools used based on the response. Our survey found varying levels of satisfaction, 64.3% of clinical governance leaders described their tool as ‘very helpful’ to ‘somewhat helpful’ in helping them to understand local healthcare OC.

Only 2% of trusts told us they were using more than one tool to measure culture, suggesting that clinical governance leaders are expecting that the tool they use will accurately report on all aspects of culture, however on reviewing the tools in use it is clear, for the majority, that the main focus remains on patient safety culture.

A wide range of the tools reported to be used to measure healthcare OC have a very strong focus on safety culture. It is interesting that the most popular tool, to measure OC used by clinical governance leaders who responded is the national staff survey. The survey covers areas such as morale, quality of care, and safety culture, which we know can be affected by workers perceptions of OC [24]. The survey is primarily designed to enable trust leaders to improve local working conditions and practices and to increase involvement and engagement with staff [24]. We found that clinical governance leaders were becoming increasingly concerned with staff morale and how staff were supported in practice; this is something that the most popular tool captures.

This study updates a study conducted by Mannion et al. [13] We found that in the last decade, despite an increased focus and understanding on culture and its impact on patient care, tools being used to measure culture have changed surprisingly little. Mannion et al. [13] found that the Manchester Patient Safety Questionnaire (MaPSaF) was the most common tool being used in 2008, and data from this study shows that MaPSaF is still in the top three.

MaPSaF was among one of the lowest rated tools in terms of satisfaction, despite being well used. This data could suggest that the tool has fallen out of favour with those who responded to the survey. The tool rated highest in terms of satisfaction was the staff participation engagement and communication application, which was rated five out of five. Although it should be noted that only one trust currently reported using this tool, and that satisfaction is subjective. In the qualitative comments expressed about this tool, its strengths included that it was an online application that could provide live data feedback.

The increased focus on patient safety meant that very few of the tools look to question the determinants of healthcare OC. Work by McSherry and Beardsmore [25] found that there were two statistically significant factors in determining healthcare OC—‘Professional practice and support’ and ‘Workforce and service delivery’. Each of these factors contains two determinants as shown in Table 3.

As part of the study we asked clinical governance leaders if they felt that the tool which they were currently using was missing any aspect of culture. Of the respondents that answered the question, 42% told us that incivility/bullying and rudeness was something that the current tools did not capture but was a high priority for them in their organisation.

This was an interesting finding, as the discourse about the impact of incivility in healthcare practice is relatively new [26]. It is clear from this research that the impact that this is having is known anecdotally by clinical governance leaders. There is a developing body of literature to support the view that incivility is impacting on healthcare delivery [27]. The authors acknowledge that in healthcare, OC and team behaviour are closely linked, and suggest that this is an area where more research is needed to understand how the culture of an organisation impacts on levels of incivility among staff.

## 5. Conclusions

Despite an increase in the number of serious incidents attributed to poor healthcare OC this has not been matched by an increase in the way NHS trusts are measuring culture. An area that is not truly understood, or widely discussed in the literature yet, is how staff experience the process of culture measurement and subsequent culture change, the authors acknowledge that this is an area that needs further research.

Recommendations for Practice

This research demonstrates that there is still a strong focus on patient safety culture. It could be that the increased focus in this area means that researchers and healthcare managers are not focusing enough on understanding the determining factors of OC. The tools that are identified under the global theme of ‘measuring OC’ do go some way to helping healthcare organisations understand the determining factors of their culture. Perhaps it is time for healthcare organisations to start using more than one tool, as it is clear from this research that there is no one size fits all tool.

The responses from this study demonstrate that incivility and rudeness in healthcare organisations is something that is of increasing concern for clinical governance leaders. We recommend that further research takes place in this area to understand how healthcare OC impacts on incivility and rudeness in teams.

## Figures and Tables

**Table 1:** Tools currently in use by respondents.

Name of Tool	Number of Trusts Using Tool	Mean Average Satisfaction Score (1–5 *)	Brief Description of Tool	Global Theme
Annual Staff Survey	17	4	Online 31 question survey collecting staff views about working in their NHS organisation. Data collected about local working conditions for staff, the survey is administered annually.	Measures Organisational culture.
Friends and Family Test	14	3	Feedback tool completed by patients. Asks if patient would recommend the services they have used and offers a range of responses.	Measures Satisfaction.
Manchester Patient Safety Questionnaire	10	2	A team-based self-reflection tool that helps teams to recognise that patient safety is a multidimensional concept.	Measures Patient safety culture.
Culture and Leadership Programme	8	3	Whole programme is made up of three phases, phase one specifically looks at measurement, defined by the tool as discovery phase. Phase one measures current culture using existing data, board, staff, and stakeholder perceptions and knowledge, and workforce analysis. Before the next phase of the tool guides trusts in how to target the right areas for compassionate and inclusive leadership strategy.	Clinical governance strategy.
Culture of Safety: The Safety Attitudes Questionnaire	8	3.25	An internationally used survey that seeks to assess the attitudes toward safety in a variety of clinical settings. Data is shared with other organisations so that they can benchmark against similar organisations.	Measures Patient safety culture.
Culture of Care barometer	5	4	Online or paper-based questionnaire, designed to help organisations measure the culture of care they provide. It is a self-assessment diagnostic tool that can be used to stimulate reflection and understanding of the culture of care.	Measures Organisational culture.
Audit and appraisal	4	2	This is not a specific tool, although four trusts told us they were using locally agreed appraisal standards to measure culture in their organisation.	Inspection/audit
Incident reporting	3	3	Not a specific tool, although three trusts told us they are measuring their organisations culture through the reporting of incidents/near misses.	Inspection/audit
Care Quality Commission (CQC) inspection reports	3	3	Not a specific tool, although three trusts told us they are measuring their organisations culture through CQC reports. Inspections focus on five domains: Are they safe? Are they effective? Are they caring? Are they responsive to people’s needs? Are they well-led?	Inspection/audit
Affina Organisational development Team development tool	2	2.5	Commissioned organisation that offers trusts a range of services from team assessment to cross-system team development. Trusts are then given the responsibility for development, with support provided.	** unable to categorise as questionnaire not available to researchers
Values and behaviours programme	2	4	Local (trust based) programme to engage staff members in understanding and embodying the values and behaviours of that organisation.	Clinical governance strategy.
Cultural web	2	3.5	The Cultural Web identifies six interrelated elements: Stories, rituals and routines, symbols, organisational structure, control systems and power structure. These elements are reviewed to look at how they are impacting on the culture of that organisation. The cultural web can be used to assess current culture and also to analyses where the organisation wants to be.	Measures organisational culture.
AOD culture assessment tool	2	3.5	This is a commissioned tool to assess the ways in which an organisation is working well, as well as the areas that need to change.	Measures organisational culture.
Fab O Meter	2	2.5	Staff are asked daily to record ‘How was work today?’ answers are recorded via an app. Data creates a picture of morale and can be split into professions and departments in individual organisations. Can also be used to benchmark against national and regional performance.	Measures morale.
Staff Participation Engagement and Communication Application.	1	5	Also known as the ‘Happy App’. The app collects data from staff about problems and frustrations as well as positive experiences. Managers can access data in real time to address issues.	Measures morale
Medication safety culture tool	1	4	Medication safety climate questionnaire for healthcare staff in UK hospitals. 50 item questionnaire focusing on nine factors: teamwork climate, safety climate, job satisfaction, stress recognition, perceptions of management, working conditions, organisational learning, feedback and communication about error, and management support for medication safety.	Measures patient safety culture.

* Clinical governance leaders were asked to score tools based on satisfaction, one being unsatisfied, five being very satisfied. ** Affina Organisational development Team development tool authors were asked to provide the full tool, however this was not made available.

**Table 2:** Global themes defined.

Theme	Definition.
Measures Organisational culture	Tool has supporting literature that suggests that the tool confidently measures aspects of organisational culture.
Measures Satisfaction	The tool does not measure organisational culture, it measures satisfaction with services delivered.
Measures Patient Safety Culture	The tool does not measure organisational culture, it focuses on patient safety culture and/or climate.
Measures Morale	The tool does not measure organisational culture, it measures staff morale at a given moment in time.
Inspection/Audit	Not a specific tool.
Clinical Governance strategy	Not a specific tool.

**Table 3:** Determinants of organisational culture [25].

**Factor 1—Professional Practice and Support.**	
Determinant 1: Professional practice. Staff feel able to carry out care to their satisfaction. Staff are supported to undertake professional development. Staff are involved in multi-disciplinary team meetings. Staff feel discharge planning is done effectively. Hand hygiene is carried out to a high standard.	Determinant 2: Support. Appraisals and personal development take place. Clinical areas reliance upon bank/ agency nursing staff. Actions are followed up.
**Factor 2—Workforce and service delivery.**	
Determinant 3: Workforce. Planned study leave is cancelled. There is a shortage of nursing staff in the clinical area. There is a high turnover of nursing staff. There is a high sickness absence rate.	Determinant 4: Service delivery. There are unplanned re-admissions following discharge. There is a shortage of medical staff in the area. The clinical area looks untidy. Patients and relative make complaints.
